# Melatonin rhythm disorder is more pronounced in major depressive disorder with non-suicidal self-injury

**DOI:** 10.3389/fnins.2025.1534715

**Published:** 2025-05-13

**Authors:** Xiaojuan Hu, Aiguo Zhang, Chao Wang, Xulai Zhang

**Affiliations:** ^1^School of Mental Health and Psychological Science, Anhui Medical University, Hefei, China; ^2^Department of Psychiatry, Anhui Medical University Affiliated Psychological Hospital, Hefei, China; ^3^Anhui Provincial Mental Health Center, Hefei, China; ^4^The Fourth People’s Hospital of Hefei City, Hefei, China

**Keywords:** non-suicidal self-injury, melatonin, circadian rhythm, ottawa self-injury scale, depression

## Abstract

**Introduction:**

Disruptions in melatonin (MT) rhythms have been linked to major depressive disorder (MDD) and may be further associated with non-suicidal self-injury (NSSI). This study investigates whether MDD patients with NSSI exhibit more pronounced MT rhythm disturbances and whether these disruptions correlate with NSSI-related thoughts and the motivation to cease self-injury.

**Methods:**

The study included 100 participants aged 14–24 years, including 30 healthy controls (HC) and 70 inpatients diagnosed with MDD. The MDD group was further divided into those with NSSI (NSSI group, *n* = 35) and those without NSSI (Non-NSSI group, *n* = 35). Salivary MT levels were measured at six intervals (12 a.m., 8 a.m., 11 a.m., 1 p.m., 4 p.m., and 10 p.m.) using enzyme-linked immunosorbent assay (ELISA). The Ottawa Self-Injury Inventory (OSI) assessed NSSI perception and motivation in the NSSI group.

**Results:**

Melatonin levels were significantly lower across all six time points in the NSSI group compared to both the Non-NSSI and HC groups (*P* < 0.05), and MT circadian rhythms were notably absent in the NSSI group. Correlational analysis revealed specific associations between MT levels and NSSI behavior, with MT levels at 1 PM positively correlated with invasive self-harm impulses (r = 0.487, *P* = 0.003, 95% CI: 0.141 to 0.834) and MT levels at 8 a.m. and 11 a.m. inversely correlated with the desire to stop self-injury (r = −0.427, *P* = 0.010, 95% CI: −0.774 to −0.081; r = 0.348, *P* = 0.040, 95% CI: 0.002 to 0.695, respectively).

**Conclusion:**

Lower MT levels and disrupted circadian rhythms are associated with NSSI in MDD patients, highlighting a potential link between circadian dysfunction and self-injurious behaviors. Further research is needed to clarify the underlying mechanisms of this association.

## Introduction

Major depressive disorder (MDD) is a mental health condition marked by persistent low mood and other debilitating symptoms. Among adolescents, MDD often co-occurs with non-suicidal self-injury (NSSI) (Plener et al., 2015). NSSI includes various socially unacceptable behaviors where individuals intentionally harm their own bodies without suicidal intent (Schneiderman et al., 2005). Previous studies have shown a strong association between MDD and NSSI, with MDD serving as a major risk factor for NSSI. Depression, impulsivity, and suicidal ideation are significantly correlated with NSSI, and these symptoms are more pronounced in MDD patients who engage in self-injury (Kinyanda et al., 2005; Rodav et al., 2014). Notably, NSSI frequently overlaps with suicidal behaviors; patients with NSSI have a suicide rate 100 times higher than the general population in the following year and continue to exhibit elevated suicide rates over time, with more than 5% ultimately committing suicide within 9 years (Asarnow et al., 2011; Cowen et al., 2012). Consequently, NSSI, in addition to established risk factors like depressive symptoms and suicidal tendencies, is a critical factor for assessing and predicting suicide risk in adolescents (Guan et al., 2012; Tuisku et al., 2014).

Delayed circadian rhythms in adolescence and early adulthood have been linked to the severity of depressive and emotional symptoms (Lewy, 2010; Raniti et al., 2017), and circadian rhythm disturbances put adolescents at a higher risk of mental illness (Addington and Heinssen, 2012). Various circadian rhythm disruptions—such as alterations in sleep-wake cycles and irregular secretion of melatonin (MT) and cortisol—are also associated with depressive symptoms (Abreu and Bragança, 2015). Cavallo et al. (1987) reported that 24 h MT concentrations in adolescents aged 7–15 with depressive symptoms were lower than in those without depression. Similarly, a Swedish study found a negative correlation between nighttime MT levels and depressive symptoms in individuals aged 18–25, indicating that lower nighttime MT secretion corresponded with more severe depressive symptoms (Sundberg et al., 2016). Another study by Robillard et al. (2017) linked delayed MT rhythms and reduced myo-inositol concentration in the anterior cingulate cortex to depression in adolescents. However, a contrasting United States study found that adolescents aged 8–17 with depressive symptoms had higher nighttime MT levels than their non-depressed counterparts (Shafii et al., 1996).

This study aims to investigate whether MDD patients with NSSI exhibit more severe MT rhythm disorders and abnormal MT levels and to explore the potential association between these abnormalities and NSSI.

## Materials and methods

### Participants

The study sample consisted of 70 hospitalized patients aged 14–24 who met the DSM-V-TR diagnostic criteria for MDD. These participants were admitted for treatment at the Affiliated Psychological Hospital of Anhui Medical University between 2020 and 2023. All patients had no history of head injury, severe physical or neurological disorders, or psychoactive substance abuse. Additionally, participants were required to refrain from using exogenous melatonin or other sleep-related supplements for at least 2 weeks prior to data collection, and patients who had taken SSRIs or other psychotropic medications within the past 4 weeks were excluded to minimize pharmacological influences on endogenous melatonin levels and circadian rhythm. Female participants were excluded if they were pregnant or lactating. The patients were divided into two groups: those with non-suicidal self-injury (NSSI group) and those without NSSI (Non-NSSI group). Healthy controls were recruited from the general population and were comparable to the MDD group in terms of age and gender. All controls were screened to exclude psychiatric illness and sleep-related disturbances. Besides, light exposure and sleep schedule were strictly controlled during the study. All participants followed a standardized sleep–wake schedule, going to bed at around 9:00 p.m. and waking up at around 6:00 a.m. Nighttime saliva samples were collected at the bedside under dim light conditions, and participants were instructed to avoid using electronic devices for at least 2 h before sleep. Prior to daytime sample collection, participants stayed in a dimly lit room for at least 30 min to minimize the influence of ambient light.

### Clinical evaluation

The clinical evaluation was independently conducted by two experienced attending psychiatrists, using the Hamilton Depression Rating Scale (HAMD) to assess disease severity.

### Measurement of saliva MT levels

Prior to sample collection, participants were instructed to follow these precautions: refrain from eating for at least 1 h, avoid brushing teeth within the past 10 min, and abstain from drinking water. Samples were collected at six time points: 0 a.m., 8 a.m., 11 a.m., 1 p.m., 4 p.m., and 10 p.m. Saliva was collected using saliva cups. A cotton swab was placed under each participant’s tongue for approximately 3–5 min, then returned to the saliva cup and stored in a refrigerator (0°C–4°C) within 30 min of collection.

Each sample was centrifuged at 3,000 rpm for 15 min, and the resulting saliva was stored at −20°C for 4 h before further analysis. Salivary MT concentration was measured using enzyme-linked immunosorbent assay (ELISA), following the manufacturer’s instructions.

Equal portions of each sample were used for MT level evaluation, with each specimen measured at least twice by the same researcher. The intra- and inter-batch coefficient of variation for salivary MT was less than 2.5%, with levels expressed in pg/mL.

### Evaluation of the ottawa self-injury inventory (OSI)

The OSI (Martin et al., 2013; Nixon et al., 2015) is a 28-item self-report questionnaire designed to comprehensively evaluate the cognitive, emotional, behavioral, motivational, and environmental aspects of NSSI. The OSI provides quantitative ratings of self-harm behaviors along with qualitative insights into motivational factors underlying NSSI. For our research objectives, we focused on the following items:

No. 7: “When you experience self-harm impulses, how do these impulses feel to you?” (Options: Pain or chaos, Comfort, Invasive or aggressive). Each participant rated three possible impulse types on a scale from 0 (not at all) to 4 (extremely obvious).

No. 24: “When you try to resist self-harm behavior (non-suicidal), how motivated are you to stop?” Scores ranged from 0 (not motivated at all) to 4 (extremely motivated).

### Statistical analysis

The data analysis was performed using SPSS 22.0 statistical software. For continuous data that follow a normal distribution, group comparisons were conducted using analysis of variance (ANOVA) or *T*-test. For comparisons of MT levels at different time points between groups, repeated measures ANOVA was used. As this was an exploratory study with a relatively small sample size, no formal correction for multiple comparisons was applied. Findings should therefore be interpreted with caution. And no formal a priori power analysis was conducted. The sample size was based on the number of eligible participants available during the data collection period. Categorical data was presented as rates (%) and group comparisons were conducted using a χ2 test. The correlation analysis between two variables was performed using Pearson correlation. All figures in this study were generated using R (4.3.1). A two-tailed probability was used for calculating *p*-values, with *P* < 0.05 indicating statistically significant differences.

## Results

### Demographic and clinical characteristics

This study included 35 cases in the NSSI group (MDD patients with NSSI), 35 cases in the Non-NSSI group (MDD patients without NSSI), and 30 cases in the Healthy control group (HC group). Demographic and clinical characteristics of three groups of participants summarized in Table 1.

**TABLE 1:** Three groups of demographic and clinical characteristics.

Variable		NSSI group (*n* = 35)	Non-NSSI group (*n* = 35)	HC group (*n* = 30)	Cohen’s d	*P-*value (NSSI Vs non-NSSI)
Age (years)		18.20 ± 2.85	18.14 ± 2.68	19.17 ± 2.31	0.02	0.228
Education (years)		10.60 ± 2.38	10.80 ± 2.65	11.70 ± 2.09	–0.08	0.157
Gender [*n* (%)]	Male	16 (45.71)	15 (42.86)	15 (50.00)	–	0.846
	Female	19 (54.29)	20 (57.14)	15 (50.00)		
BMI (kg/m^2^)		20.69 ± 1.90	21.14 ± 1080	20.83 ± 2.18	–0.29	0.616
HAMD		18.40 ± 4.30	19.14 ± 4.39	–	–0.17	0.434

BMI, body mass index; HMAD, Hamilton Depression Scale.

Data are presented as mean ± standard deviation (SD) or *n* (%). *P*-values were calculated using *T*-test for continuous variables and chi-square test for categorical variables (gender). HAMD scores were compared between NSSI and Non-NSSI groups using an independent samples *t*-test.

### Group comparisons of saliva MT levels and circadian rhythm

**FIGURE 1:**
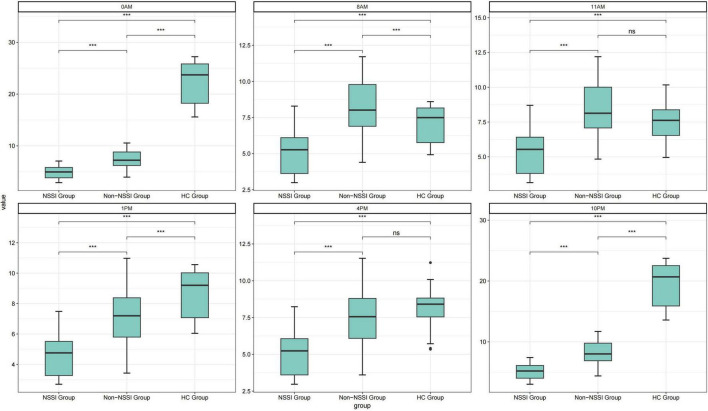
Comparison of salivary melatonin (MT) levels across six time points among non-suicidal self-injury (NSSI), Non-NSSI, and healthy controls (HC) groups. ***p < 0.001.

**FIGURE 2:**
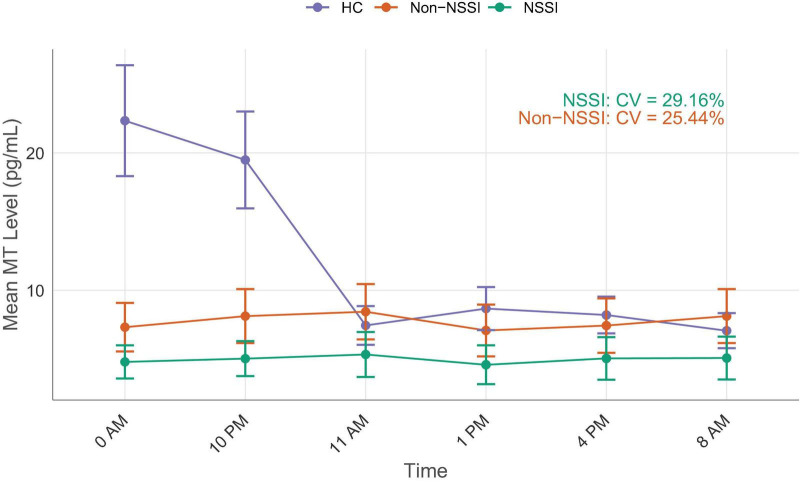
Changes in melatonin (MT) Levels across different time points in non-suicidal self-injury (NSSI), Non-NSSI, and healthy controls (HC) groups. Error bars represent SD.

The salivary MT levels in the NSSI group decreased significantly at six time points compared to the other two groups (Figure 1). The MT levels in the control group showed a significant diurnal rhythm trend, with lower levels during the day and higher levels at night. However, this circadian rhythm disappeared in the other two groups (Figure 2).

**FIGURE 3:**
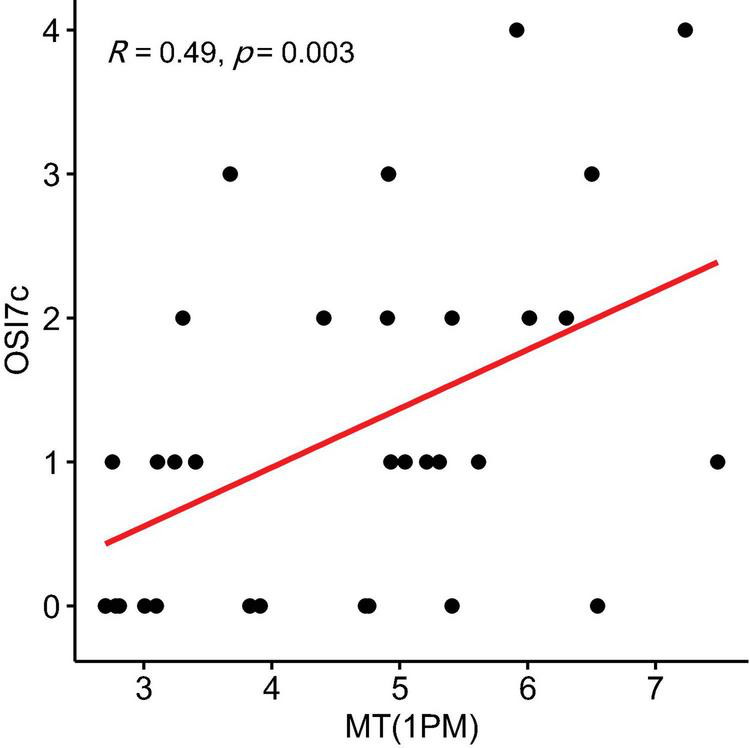
Correlation Between melatonin (MT) levels at 1 p.m. and OSI7c scores (invasive or aggressive self-injury) in the non-suicidal self-injury (NSSI) Group. The shaded area around the regression line represents the 95% confidence interval.

**FIGURE 4:**
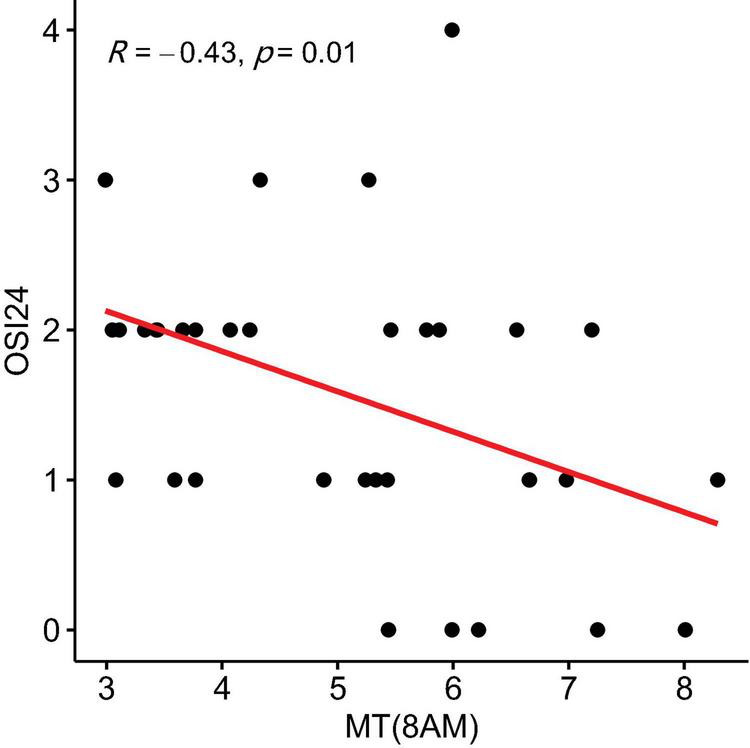
Correlation Between melatonin (MT) Levels at 8 a.m. and OSI24 scores (motivation to cease self-injury) in the non-suicidal self-injury (NSSI) Group. The shaded area around the regression line represents the 95% confidence interval.

**FIGURE 5:**
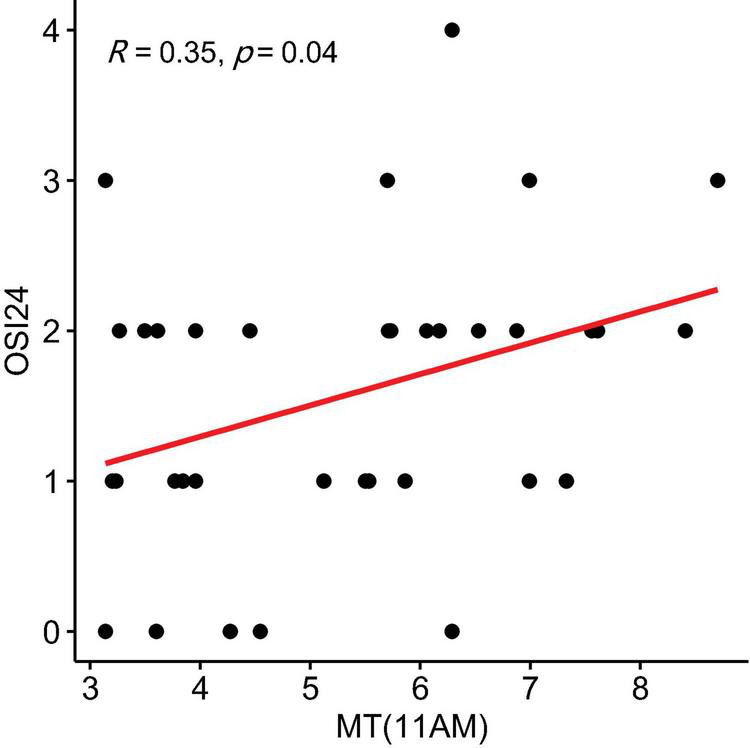
Correlation Between melatonin (MT) Levels at 11 a.m. and OSI24 Scores (motivation to cease self-injury) in the non-suicidal self-injury (NSSI) Group. The shaded area around the regression line represents the 95% confidence interval.

In the NSSI group, OSI7c (self-injury as invasive or aggressive) showed a positive correlation with MT levels at 1 p.m. (r = 0.487, *P* = 0.003, 95% CI: 0.141 to 0.834) (Figure 3). OSI-24 (motivation to cease self-injury) was negatively correlated with MT levels at 8 AM (r = −0.427, *P* = 0.010, 95% CI: −0.774 to −0.081) (Figure 4) and positively correlated with MT levels at 11 AM (r = 0.348, *P* = 0.040, 95% CI: 0.002 to 0.695) (Figure 5).

## Discussion

This study reveals a substantial connection between MT rhythm disturbances and the presence of NSSI in patients with MDD. Our findings suggest that the abnormal circadian rhythm and significantly lower levels of MT in the NSSI group reflect an underlying dysfunction of the hypothalamic-pituitary-adrenal (HPA) axis. The HPA axis, responsible for managing stress responses, appears to be excessively activated in NSSI patients, likely due to the demands of chronic psychological stress. This excessive activation could disrupt MT levels through negative feedback mechanisms, directly influencing the motivation to engage in self-injurious behavior. More specifically, chronic activation of the HPA axis in MDD patients—especially those with NSSI—leads to sustained elevations in cortisol levels. Elevated cortisol can suppress pineal gland activity and downregulate the expression of arylalkylamine N-acetyltransferase (AANAT), the rate-limiting enzyme in melatonin synthesis (Gozdowska et al., 2022). Furthermore, excessive cortisol may impair the function of the suprachiasmatic nucleus (SCN), the master circadian pacemaker, by disrupting glucocorticoid receptor-mediated signaling pathways (Zhang et al., 2014). These effects collectively result in diminished melatonin synthesis and loss of circadian rhythmicity, as observed in our NSSI group.

In line with previous research indicating that MT has neuroprotective, anti-inflammatory, and stress-regulating properties (Che et al., 2020; Ahmed et al., 2021; Cho et al., 2021; Madebo et al., 2021). Our findings add to the growing body of evidence indicating that melatonin dysregulation is associated with greater depressive symptom severity and self-injurious behavior. The reduced MT levels and disrupted diurnal rhythm observed in the NSSI group may reflect underlying circadian misalignment and HPA axis disturbances, which have been previously linked to impaired emotional regulation. While causality cannot be established from this cross-sectional design, these associations highlight the need for further investigation into the neurobiological pathways connecting circadian rhythms and maladaptive coping behaviors.

A noteworthy finding in this study is the correlation between specific MT levels at different times of the day and distinct aspects of self-injurious behavior. For example, MT levels at 1 p.m. showed a positive correlation with aggressive and invasive self-harm impulses, suggesting that fluctuations in MT may be related to the intensity and nature of self-injury urges. Furthermore, MT levels at 8 a.m. and 11 a.m. were associated with the motivation to stop self-injury, reflecting the complex interplay between circadian rhythms, emotional regulation, and behavioral control. The significance of these correlations at specific time points may reflect the interaction between melatonin and cortisol rhythms. Under normal physiological conditions, cortisol peaks around 8 a.m. and then declines. In MDD patients with NSSI, this rhythm may be dysregulated or persistently elevated. Interestingly, our findings suggest that higher melatonin levels at 8 a.m. are positively associated with the desire to stop self-injury, which could reflect a more normalized HPA axis response and greater access to internal motivation (e.g., hope for change, relief from distress—positive feedback mechanisms). In contrast, at 11 a.m., melatonin levels were negatively associated with the desire to stop self-harm. At this time, healthy individuals typically exhibit lower cortisol levels, but in patients with persistent HPA axis hyperactivity, fear-driven or avoidance-based inhibition (negative feedback mechanisms) may dominate the decision to stop self-injuring.

The associations observed between melatonin levels and NSSI-related behaviors may be rooted in melatonin’s neurobiological roles beyond circadian regulation. Melatonin has been shown to modulate the stress response system by downregulating HPA axis activity and lowering cortisol levels (Azpeleta et al., 2024; Pachimsawat et al., 2024). It also interacts with brain areas involved in emotional regulation, such as the limbic system and prefrontal cortex, potentially contributing to mood stability and impulse control (Repova et al., 2022; Paribello et al., 2022). Additionally, melatonin’s involvement in pain perception and its documented analgesic effects may be particularly relevant, as altered pain sensitivity is a common feature in individuals who engage in NSSI (Oh et al., 2020). These pathways offer a potential mechanistic framework to explain how disrupted melatonin rhythms might influence both the urges and motivations underlying self-injurious behaviors.

### Clinical implications

The potential clinical implications of our findings are 2-fold. First, we observed that disruptions in melatonin rhythms—particularly elevated daytime levels and decreased morning levels—were more pronounced in MDD patients with NSSI, suggesting an association with heightened self-injury impulses and impaired behavioral inhibition. Although not diagnostic on their own, salivary melatonin patterns may serve as adjunctive indicators in the clinical evaluation of patients with depression and suspected NSSI. In particular, melatonin abnormalities at specific time points (e.g., elevated levels in the early afternoon) may help identify periods of increased emotional vulnerability, potentially informing the timing of therapeutic interventions.

Second, interventions aimed at restoring circadian stability—such as light therapy, sleep hygiene education, or melatonin receptor agonists—represent promising areas for future research in understanding their potential role in managing NSSI behavior in MDD populations. However, further studies are required to assess the effectiveness of these interventions in clinical practice. Importantly, we do not propose melatonin as a direct biomarker for suicide risk in all patients with depression. Rather, its utility may lie in aiding clinical decision-making in complex or ambiguous presentations, particularly when patients are uncooperative or have unclear psychiatric histories. Further longitudinal and interventional studies are warranted to clarify causal relationships and evaluate the clinical feasibility of circadian-based interventions in this population.

### Limitation

However, this study has several limitations. First, due to its cross-sectional design, causal inferences between melatonin dysregulation and NSSI cannot be made. Although we observed significant associations, the possibility of reverse causality cannot be excluded—that is, engagement in NSSI or its associated psychological distress may, in turn, disrupt melatonin secretion and circadian rhythms. Second, although we excluded patients with major psychiatric comorbidities such as bipolar disorder and schizophrenia, we did not systematically assess or control for other common comorbid conditions such as anxiety, PTSD, or trauma-related disorders. These factors may independently influence both melatonin levels and self-injurious behavior. Third, while light exposure and sleep schedules were strictly controlled throughout the study, other potential confounding variables—such as dietary factors, physical activity, and subtle medication histories—were not fully accounted for. Nonetheless, we minimized pharmacological influences by excluding participants who had used SSRIs within 4 weeks or melatonin/sleep-related supplements within 2 weeks prior to saliva collection. Future research should incorporate longitudinal and interventional designs, employ structured diagnostic assessments, and statistically control for additional lifestyle and clinical variables. Such approaches will be crucial to clarifying the directionality of the observed associations and validating the clinical relevance of melatonin dysregulation in NSSI.

## Conclusion

This study demonstrates a significant association between disrupted melatonin rhythms and NSSI in patients with MDD. The observed abnormalities in melatonin levels, particularly during the daytime, may reflect underlying dysregulation of the HPA axis and circadian misalignment, which could contribute to emotional instability and reduced behavioral control. Although melatonin patterns are not diagnostic markers on their own, they may offer supplementary value in identifying individuals with heightened emotional vulnerability. Given the cross-sectional nature of this study, no causal inferences can be drawn. Future longitudinal research, ideally involving medication-free and comorbidity-controlled samples, is warranted to clarify the directionality of these associations and to explore whether circadian-based interventions could be effective in reducing NSSI risk among MDD populations.

## Data Availability

The datasets presented in this article are not readily available because the data involves sensitive information or personal privacy. In order to protect the privacy of the participants, the data cannot be shared publicly. Requests to access the datasets should be directed to AZ, 175784411@qq.com.
